# Bioactive compound ginkgolide B alleviates food allergy by regulating mTOR signaling pathway

**DOI:** 10.3389/fimmu.2026.1893204

**Published:** 2026-08-18

**Authors:** Rongqin Zhou, Xiaotong Yang, Panpan Tan, Huan Wang, Dan Zeng, Xiaojuan Ma

**Affiliations:** 1 School of Public Health, Zunyi Medical University, Zunyi, China; 2 Laboratory of Maternal and Child Health and Exposure Science, Guizhou Provincial Department of Education, Zunyi, China; 3 Department of Allergy, Chongqing General Hospital, Chongqing University, Chongqing, China

**Keywords:** allergy, bioactive compound, food allergy, ginkgolide B, mTOR signaling pathway

## Abstract

**Background:**

The prevalence of food allergy has been rising over the past decade, but effective treatments remain limited. The purpose of this study was to investigate the effect of ginkgolide B (GB), a natural bioactive compound with well-documented anti-inflammatory and immunoregulatory properties, on a mouse model of food allergy and to elucidate the mechanism mediated by the mTOR signaling pathway.

**Methods:**

BALB/c mice were randomly divided into five groups, namely: NC group, food allergy group (Model), low-dose GB intervention group (GBL, 5 mg/kg/day), high-dose GB intervention group (GBH, 40 mg/kg/day), and a positive control group treated with food allergy herbal formula-2 (FAHF-2). At the end of the sensitization period, the mice were intervened by gavaging GB or FAHF-2. Allergy-related factors such as serum titers, cytokines in the spleen, and intestinal morphology were detected. mTOR signaling pathway in the splenocytes was also detected.

**Results:**

After treatment, the levels of serum IgE, IgG, IgG1, and IgG2a in the GBL group were significantly lower than those in the GBH and model groups. The analysis of intestinal morphology indicated that a low dose of GB and FAHF-2 alleviated food allergy more effectively than a high dose of GB. The results of the analysis of splenic cytokines and flow cytometry showed more balanced Th1/Th2 ratio and Th17/Treg ratio after GB and FAHF-2 treatment, especially for GBL, whose effect was comparable to those of FAHF-2. The expression levels of p-STAT3, p-STAT6, and GATA-3 were significantly decreased, and the expression levels of p-STAT4 and Foxp3 were significantly increased after GB and FAHF-2 treatment. Moreover, the reductions in p-mTOR, p-AKT, p-SGK1, and p-4EBP1 induced by allergic sensitization were restored in the GBL intervention group.

**Conclusion:**

GB (5 mg/kg/day) exerts a significant therapeutic effect on food allergy, possibly by regulating the mTOR signaling pathway.

## Introduction

Food allergy refers to the adverse reactions following food ingestion, mediated by the immune mechanisms, leading to primarily dermal, respiratory, and gastrointestinal symptoms, and in severe allergic cases, patients are at risk of developing anaphylactic shock, which may ultimately result in death (1). Food allergy is broadly classified into three categories, including IgE-mediated immediate hypersensitivity, non-IgE-mediated reactions, and immune pathways co-mediated by IgE and non-IgE, dominated by the IgE-mediated type (2). The prevalence of food allergy differs between children and adults. In children, recent studies report that the incidence of diagnosed food allergy ranges from 2.53% to 7.8%, and the prevalence of food allergy in adults ranges from 0.3% to 10.8% among different countries (3–8). Alarmingly, the prevalence of food allergy has gradually increased over the past two decades, but there is no radical, universal curative therapy that can permanently eliminate food allergy symptoms for all patients at present. Food allergy has been listed as the sixth most significant public health problem in the 21st century by the WHO, and it is also considered to be one of the important reasons endangering children’s health.

For patients with food allergy, a strict elimination diet is commonly recommended. However, in practice, cross-reactive foods exist and accidental ingestion remains a concern (9). In case of food allergy, adrenaline, diphenhydramine hydrochloride, or cetirizine are generally administered based on the severity of the clinical symptoms (10). Nevertheless, these medicines can only alleviate the corresponding symptoms of the allergic reaction, while once foods containing the offending allergens are ingested, symptoms are likely to recur. Thus, components targeting at immunity imbalance of the food allergy patients are badly needed.

Traditional Chinese medicine (TCM) may serve as a promising therapeutic option for the treatment of food allergy. At present, the reported TCM formulations for food allergy include formula-1, formula-2, formula-3, EBF2, etc., among which food allergy herbal formula-2 (FAHF-2) has been internationally recognized (11). It is composed of nine herbs, including *Ganoderma lucidum*, Wumei, Chuanjiao, Guizhi, Huangbai, Huanglian, Ganjiang, Danggui, and ginseng. In a double-blind, randomized, placebo-controlled trial, 68 patients with common food allergies were enrolled, of whom 46 received FAHF-2 and 22 received placebo. No serious adverse events occurred during the treatment. After 6 months of therapy, patients receiving FAHF-2 exhibited significantly lower IL-5 and increased Treg cell counts, indicating that FAHF-2 is a safe and effective herbal medication for food allergy treatment (12). Like many TCM formulas, FAHF-2 has the drawback of inconvenient administration. Further analysis of the formula identified berberine as its effective medicinal ingredient. However, the bioavailability of berberine monomer is low, and other substances in the formula are needed to assist in its absorption, thereby limiting its standalone therapeutic use (13).

With the deepening of research on active ingredients in TCM and plant-derived foods, if a single active ingredient can intervene in food allergy, it could overcome the inconvenience of using TCM formulas. Ginkgolide B (GB, PubChem CID: 6324617) is one of the bioactive ingredients in *Ginkgo biloba*-related products, such as ginkgo dark tea and ginkgo kernel juice. With a molecular formula of C_20_​H_24_O_10_ and a molecular weight of 424.4 Da, GB is a lipophilic diterpenoid trilactone with a unique cage-like hexacyclic skeleton that bears three lactone rings and multiple hydroxyl groups (14). It has been reported to possess many pharmacological effects including anti-oxidation, anti-apoptosis, anti-shock, anti-inflammation, and anti-cognitive function decline, and it is now widely used in the clinical treatment of thrombosis, acute pancreatitis, and cardiovascular diseases (14, 15). GB was found to significantly reduce the levels of Th2-type cytokine IL-5 and eosinophil counts by downregulating T cell activation in the context of asthma treatment, suggesting that it has the potential to inhibit allergic reactions (16). However, whether GB exerts alleviating effects on food allergy and the mechanism by which it mediates anti-allergic reactions have yet to be reported. Therefore, the present study aims to investigate the effect of GB on food allergy and its underlying mechanism.

## Materials and methods

### Chemicals

Ginkgolide B, BCA protein kit, and red blood cell lysis buffer were purchased from Solarbio Life Sciences (Beijing, China). FAHF-2 was supplied by the Affiliated Hospital of Zunyi Medical University. Cholera toxin (CT) and OPD were purchased from Sigma-Aldrich (St. Louis, MO, USA). FITC-labeled anti-mouse CD4, APC-labeled anti-mouse CD25, APC-labeled anti-mouse IFN-γ, PE-Cyanine7-labeled anti-mouse IL-4, PE-labeled anti-mouse IL-17A, and PE-Cyanine5-labeled anti-mouse Foxp3 antibodies, as well as the IFN-γ, IL-4, IL-5, and IL-13 mouse ELISA kits, were purchased from Thermo Fisher Scientific (Waltham, MA, USA). HRP-conjugated anti-mouse IgE, IgG, IgG1, and IgG2a antibodies, phospho-STAT3 rabbit polyclonal antibody, phospho-STAT4 rabbit monoclonal antibody, phospho-STAT6 rabbit monoclonal antibody, Foxp3 rabbit polyclonal antibody, S6K1 rabbit polyclonal antibody, phospho-S6K1 rabbit monoclonal antibody, AKT rabbit polyclonal antibody, phospho-AKT rabbit polyclonal antibody, SGK1 rabbit monoclonal antibody, and phospho-SGK1 rabbit monoclonal antibody were purchased from Abcam (Cambridge, UK). GATA-3 rabbit monoclonal antibody and phospho-mTOR rabbit monoclonal antibody were purchased from Cell Signaling Technology (Danvers, MA, USA). GAPDH mouse monoclonal antibody was purchased from Proteintech (Wuhan, China). 4E-BP1 rabbit monoclonal antibody, phospho-4E-BP1 rabbit monoclonal antibody, and mTOR rabbit monoclonal antibody were purchased from Huabio (Hangzhou, China). The other reagents were from Yamei (Shanghai, China).

### Experimental animals

A total of 50 SPF-grade female BALB/c mice (6 weeks old, weighing 20 ± 2 g) were selected. The mice were acclimatized for 1 week before grouping, fed, and watered *ad libitum* at a temperature of 24°C ± 2°C and 50% ± 10% humidity in the animal room. Protocols were approved by the Institutional Animal Care and Use Committee of Zunyi Medical University (approval no (2021).2-567).

### Animal grouping, modeling, and treatment

The mice were randomly divided into five groups, namely: normal control group (NC), food allergy model group (Model), low-dose GB intervention group (GBL), high-dose GB intervention group (GBH), and positive control group treated with FAHF-2 (FAHF). All mice except those in the NC group were sensitized by gavaging egg white (5 mg) supplemented with cholera toxin adjuvant (10 μg) on days 7, 14, 21, and 28 (17). The mice in the NC group gavaged 300 μL saline.

**Figure 1:**
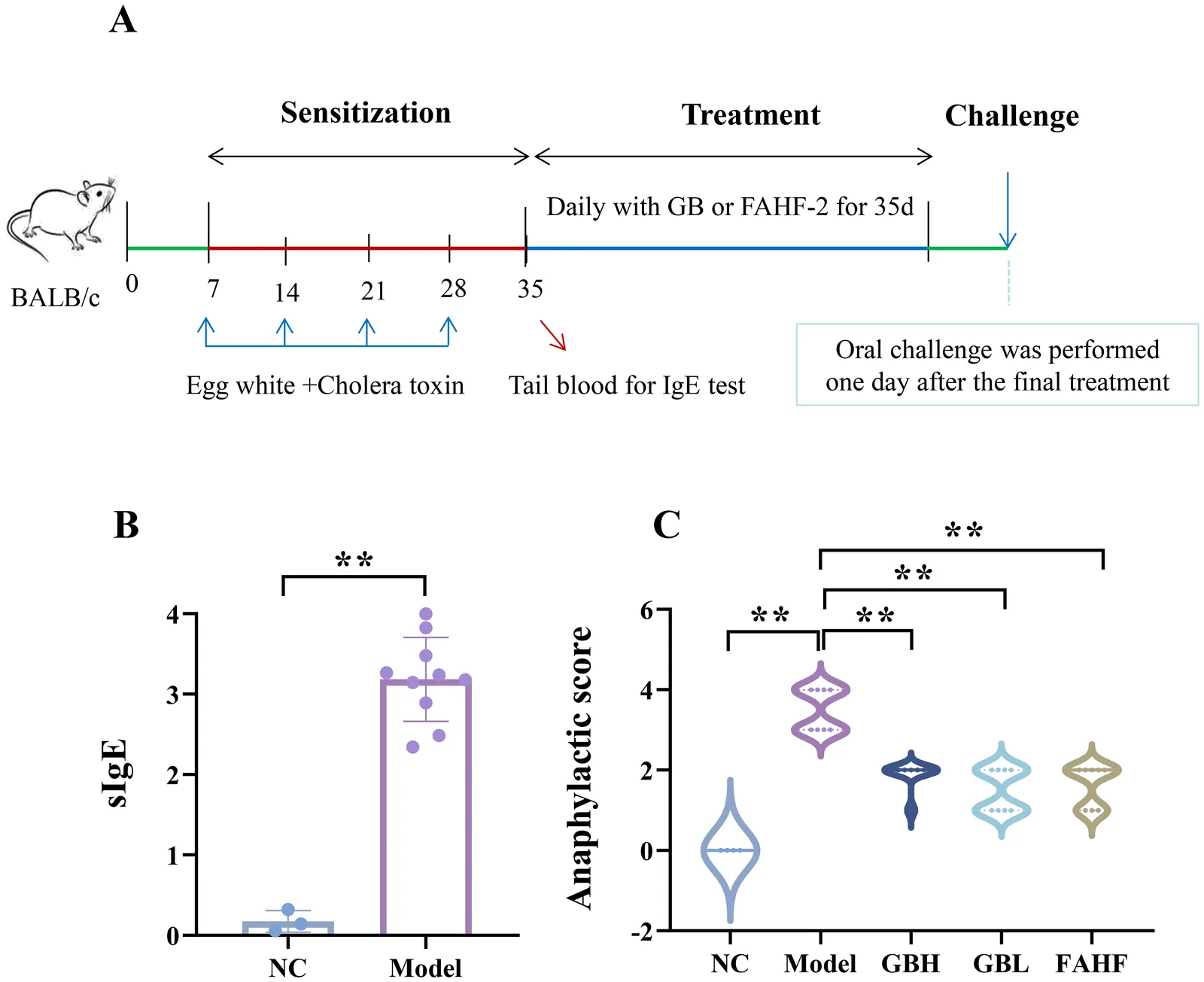
**(A)** Protocol for GB interfering with the food allergy mice model. **(B)** sIgE test on the last day of sensitization to confirm a successful model. **(C)** Anaphylactic score of the different groups of mice after challenge. **P < 0.01.

At 7 days after the last sensitization, blood was collected from the tail for sIgE detection to confirm that the mice were sensitized. Then, the GB intervention group gavaged GB daily at 5 or 40 mg/kg/day (18–20), for the GBL and GBH groups, respectively. The FAHF group gavaged FAHF-2 daily at 12 mg/day. After 35 days of treatment, all mice were challenged with 20 mg of egg white solution, and the allergic symptoms were observed 30 min after the challenge. The allergic reactions were scored as previously reported (21) with little modification, as shown in **Table 1**. Then, all mice were anesthetized and euthanized by cervical dislocation. Blood was collected for serum preparation, and the spleen and jejunum were processed as the following experiments indicated. The protocols of sensitization, treatment, challenge, and sampling are presented in **Figure 1A**.

**Table 1:** Allergic symptom score for allergic mice.

Score	Symptoms
0	Absence of symptoms
1	Scratching the nose and head, hair rising
2	Puffiness around the eyes and mouth, diarrhea, tachypnea, reduced activity or marching on the spot
3	Wheezing, dyspnea, cyanosis
4	Loss of consciousness, tremor or convulsion
5	Shock or death

### Specific IgG, IgE, IgG1 and IgG2a level detection

The titers of specific IgG, IgE, IgG1, and IgG2a were measured by indirect ELISA as previously reported (22) with minor modification: The serum was diluted at 1:5 (v:v) for IgE detection, at 1:100 (v:v) for IgG and IgG1 detection, and at 1:50 (v:v) for IgG2a detection. The secondary antibody was diluted at 1:500 (v:v) for IgE detection, at 1:1000 (v:v) for IgG detection, and at 1:2,000 for IgG1 and IgG2a detection. OPD was used for color development, and the absorbance was read at 490 nm using a microplate reader (Thermo Scientific, USA).

### Jejunum morphology observation

The jejunum was extracted, fixed in 4% paraformaldehyde for 24 h, and then dehydrated with increased concentration of ethanol. After the paraffin embedding step, the tissue was cut into 5 μm thickness and stained with hematoxylin and eosin (HE). Observation was performed with an inverted microscope (Chongqing Chongguang, China). The pathological images were then imported into the Image-Pro Plus 6.0 software, and five complete or relatively complete small intestinal villi and their associated crypts were selected for measurement.

### Detection of spleen cytokines

Mice spleens were extracted under sterile conditions and prepared and cultured as we previously reported (23). The 24-well plates were used for culture (4 × 10^6^ cells/mL). The supernatants were collected after culture for 72 h, subpackaged, and stored at −80°C until use. IFN-γ, IL-4, IL-13, and IL-17A were detected by ELISA according to the manufacturer’s instructions.

### Flow cytometry for T-cell subtype detection

The splenocytes were prepared to single cell suspension as previously described (24) and the staining process was performed according to the manufacturer’s instructions, briefly as follows: Every single cell suspension sample (1 × 10^6^ cells/mL) was divided into two parts, marking tube 1 and tube 2, and the CD4 antibody and CD4^+^CD25 mixed antibody were added and incubated for 30 min at 4°C, respectively. After washing with PBS, 500 μL fixation/permeabilization reagent was added to each tube and incubated for 30 min at 4°C. Then washing was performed and the antibodies against IL-4, IL-17A, and IFN-γ were added to tube 1, and the Foxp3 antibody was added to tube 2. After incubating at 4°C for 30 min, the labeled cells were analyzed with a Flow cytometer (Beckman Coulter, CA, USA).

### Western blot

The splenocytes were cultured as described in the Spleen cytokine detection section, and the difference is that the culture was terminated at 48 h. Then the supernatants were removed and the cells, after washing with PBS, were lysed for detection. The cell lysis and western blot steps were as previously described (25) with minor modifications: The SDS-PAGE (10% separating gel and 5% stacking gel) was carried out at 70 V for 30 min, and then changed to 120 V for 70 min. The membrane transfer was carried out under constant current of 250 mA for 180 min. Ratio of the target protein optical density value/internal reference protein GAPDH optical density value was considered as relative protein expression.

### Statistical analysis

Data in this work were analyzed with SPSS 18.0 software (IBM Corp., Armonk, NY) and results were expressed as mean ± standard deviation (SD), unless otherwise stated. Comparisons among groups were assessed by one-way ANOVA, and *P* < 0.05 was considered to be statistically significant.

## Results

### Allergic symptom score of mice after treatment

To evaluate whether intervention with GB or FAHF-2 alleviated food allergy, the successful development of mice model is a prerequisite. Therefore, before the treatment started, the specific IgE level of mice was detected by indirect ELISA. As shown in **Figure 1B**, compared with the NC group, the sIgE level of mice in the model group was significantly increased (*P* < 0.01), confirming successful model induction.

Allergic symptom changes serve as the intuitive features of treatment. As shown in **Figure 1C**, allergic symptom scores of the GBL group, the GBH group and the FAHF group were significantly lower than that in the model group (*P* < 0.01), indicating that either treatment with low dose of GB, high dose of GB or FAHF-2 worked to reduce the allergic symptoms in food-allergic mice. However, there was no statistical difference among the three treatment groups.

### Levels of specific antibodies IgE, IgG, and IgG subtypes in mice serum

**Figure 2:**
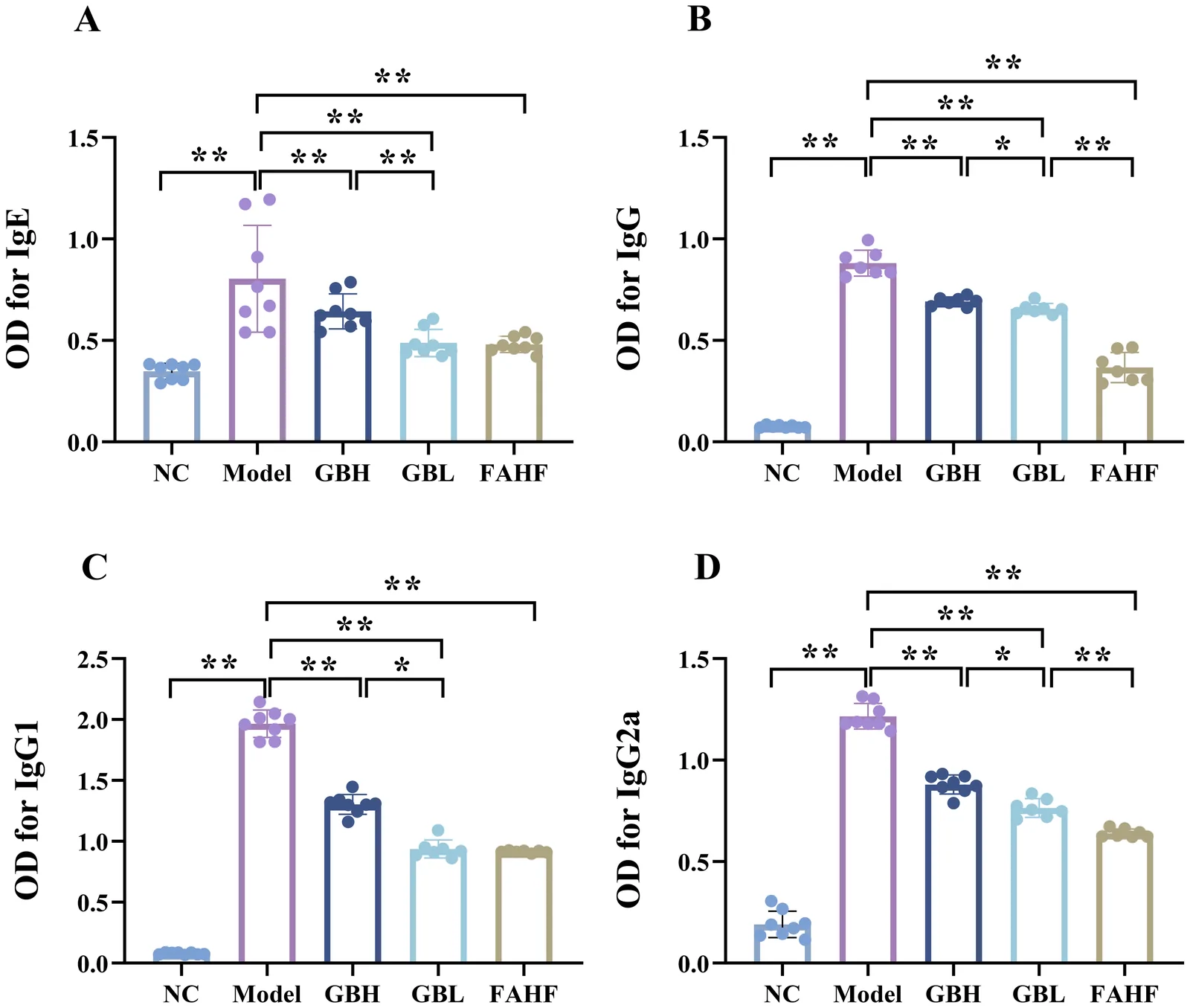
Titers of IgE **(A)**, IgG **(B)**, IgG1 **(C)**, and IgG2a **(D)** in mice serum. *P < 0.05; **P < 0.01.

Serum IgE levels in all intervention groups were lower than that in the model group (**Figure 2**), indicating that either treatment with FAHF-2, or high dose and low dose of GB effectively reduced IgE antibody of allergic mice. IgE titer in the GBL group, similar to that in the FAHF-2 intervention group, was significantly lower than that in the GBH group (*P* < 0.01), revealing that low dose of GB (5 mg/kg/day) exerted similar therapeutic effect to inhibit food allergy compared with FAHF-2, but for high dose of GB (40 mg/kg/day), the effect was not that good. For IgG, IgG1 and IgG2a antibodies, the GBL group all showed a significant decrease in OD value compared with the model group and the GBH group (*P* < 0.05).

### Effect of GB intervention on the intestinal morphology of mice

**Figure 3:**
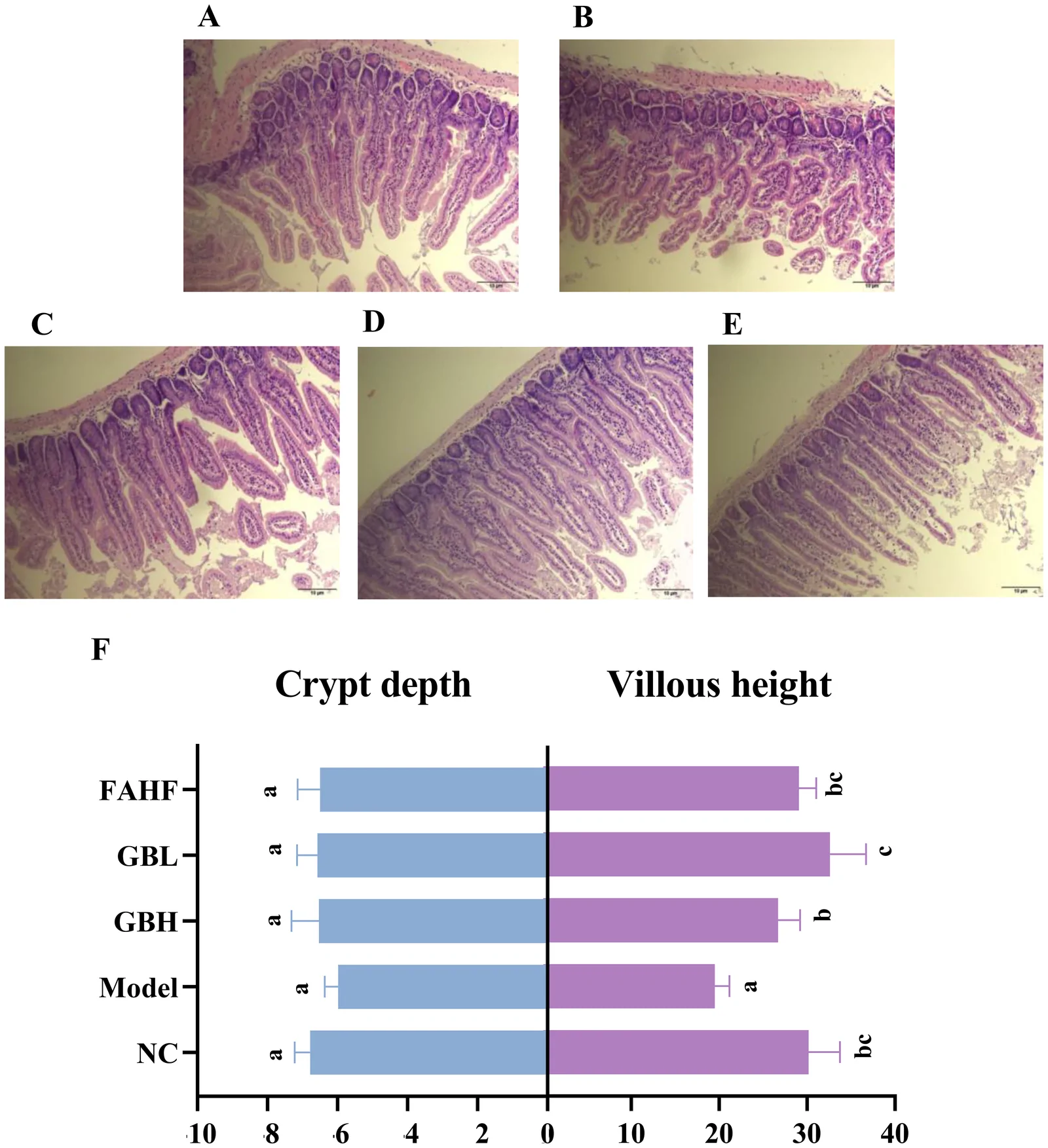
HE staining of the small intestine (400×). **(A–E)** Small intestine morphology of the blank control group, the model group, the GB 40-mg/kg/day intervention group, the GB 5-mg/kg/day intervention group, and the FAHF-2 intervention group, respectively. Different lowercase letters in **(E)** mean *P* < 0.05. Mouse crypt depth and villus height **(F)**; different letters a‑c indicate statistically significant.

Compared with the NC group, the intestinal mucosal villi in the model group exhibited marked edema, shedding, and disorganized arrangement (**Figure 3**). In contrast, the intestinal mucosal villi of the GBL group, the GBH group and the FAHF group were better organized without large localized shedding, indicating that the three treatments all worked to improve the intestinal morphology of the allergic mice. Notably, the alleviate effect was better for the GBL and the FAHF group compared with the GBH group. Villous height measurements indicated similar trends.

### Detection of mice spleen cytokines

**Figure 4:**
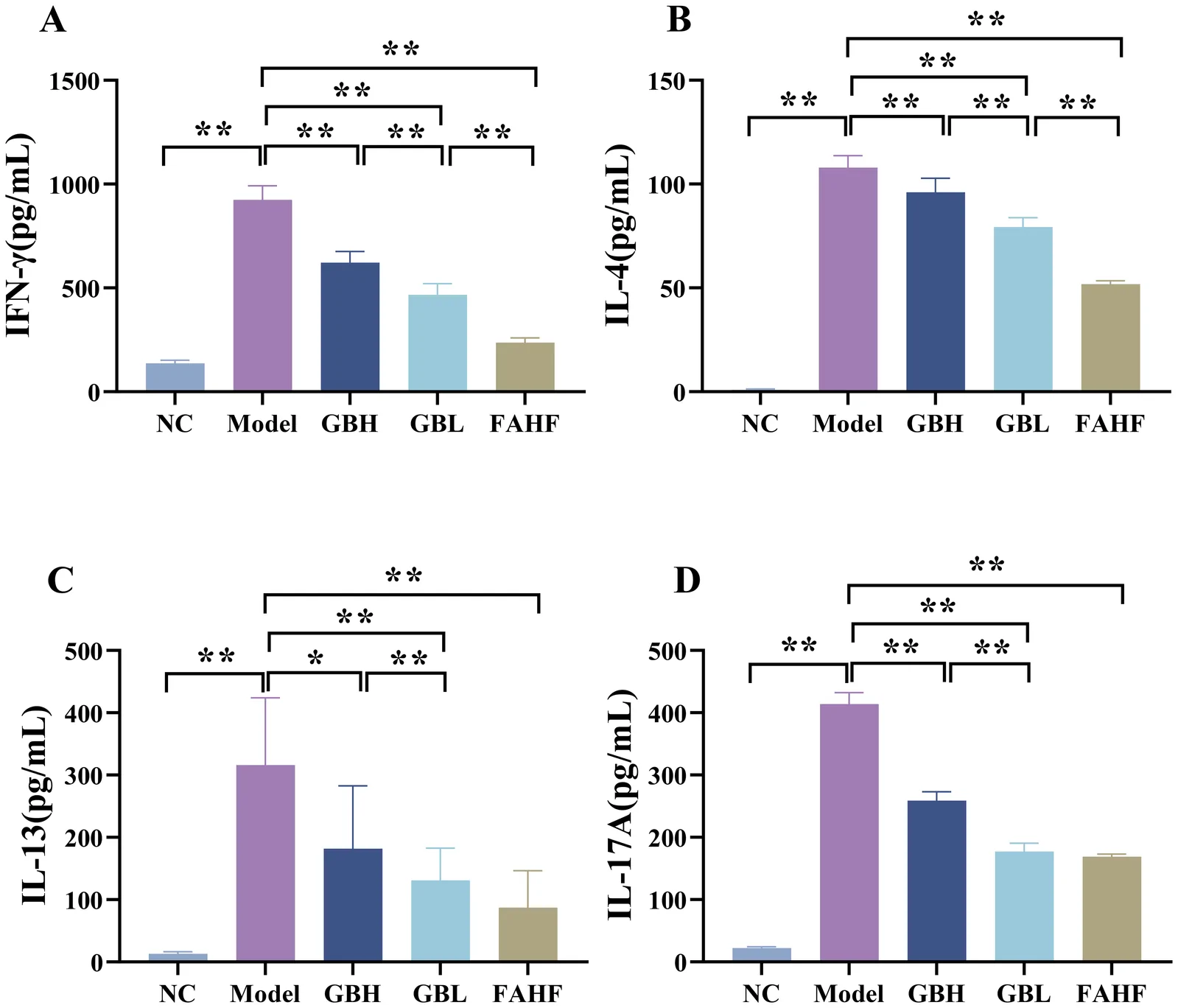
Cytokines in the splenocyte cultures. Panels **(A–D)** represent IFN‑γ, IL‑4, IL‑13, and IL‑17A, respectively. *P < 0.05; **P < 0.01.

The results of mice spleen cytokines detection are shown in **Figure 4**. IFN-γ, IL-4, IL-13, and IL-17A in each intervention group were lower than that in the model group, indicating that either treatment with FAHF-2, or high dose and low dose of GB effectively reduced spleen cytokines of the allergic mice. Compared with the GBH group, IFN-γ, IL-4, IL-13, and IL-17A in the GBL group were significantly decreased (*P* < 0.01). Furthermore, for IFN-γ and IL-4, the FAHF group was significantly lower than the GBL group (*P* < 0.01).

### Number of T cell subsets in spleen cells of mice detected by flow cytometry

**Figure 5:**
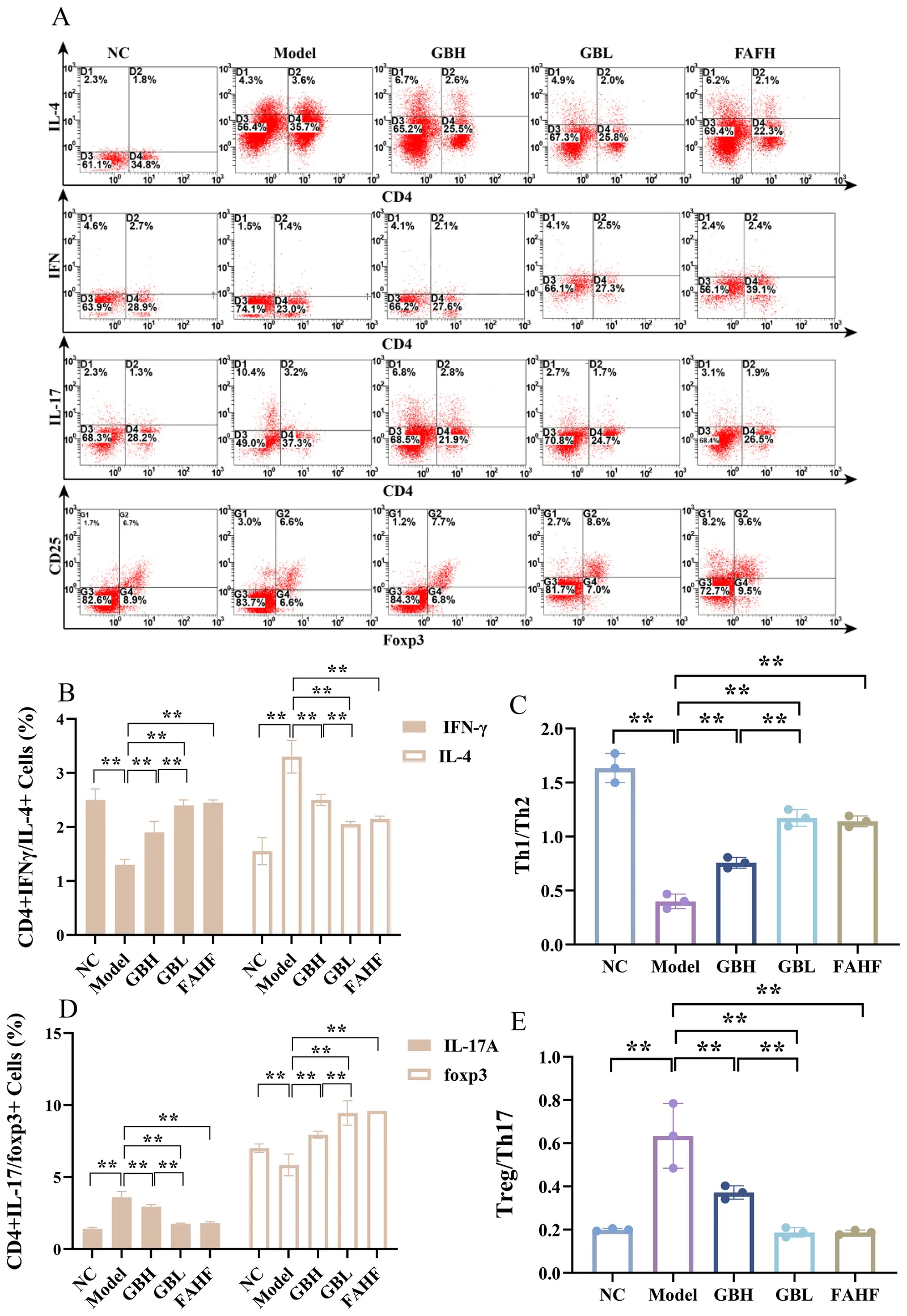
Frequencies of T cell subsets detected by flow cytometry. **(A)** Representative mouse samples in each group. **(B–E)** Percentage of T cell subtypes in each group ( $\bar{x}$ ± SEM). **P < 0.01.

The number of T cell subsets (Th1, Th2, Th17, and Treg) in the spleen of mice was determined by flow cytometry (**Figure 5**). Compared with the NC group, the ratio of CD4^+^IL-4^+^ Cells (Th2) and CD4^+^IL-17^+^ Cells (Th17) was increased in the model group, while the ratio of CD4^+^IFN-γ^+^ Cells (Th1) and CD4^+^CD25^+^foxp3^+^ Cells (Treg) was decreased. Compared with the model group, the GB treatment groups had decreased Th2 and Th17 ratio and increased Th1 and Treg ratio, which was similar to the FAHF group. Compared with the GBH group, the GBL group had decreased Th17 and Th2 ratio and increased Th1 and Treg ratio (*P* < 0.01). The Th1/Th2 and Treg/Th17 results also indicated a comparable therapeutic efficacy of GBL and FAHF-2, both of which were superior to that of GBH.

### Effect of GB intervention on the related protein of spleen cells in mice

**Figure 6:**
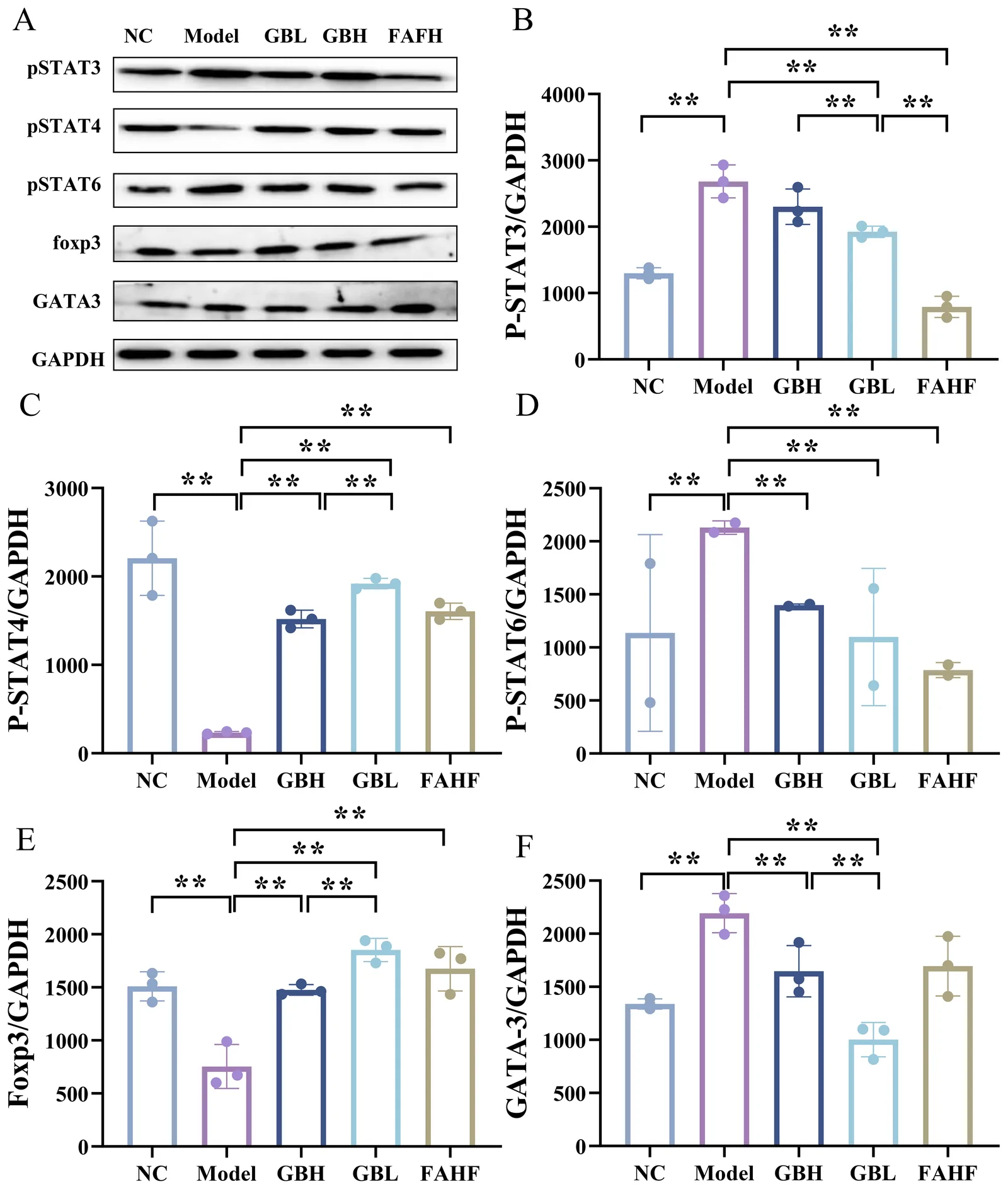
Detection of related proteins in mouse spleen cells. **(A)** Mouse protein bands in each group. **(B–F)** Expression of p-STAT3, p_STAT4, p-STAT6, Foxp3, and GATA-3 in mouse spleen cells (*n* = 5, $\bar{x}$ ± SEM). **P < 0.01.

Western Blot was used to detect the expression of mouse spleen cell-related proteins p-STAT3, p-STAT4, p-STAT6, Foxp3, and GATA-3, see **Figure 6**. Compared with the NC group, the expression levels of p-STAT3, p-STAT6, and GATA-3 in the model group were significantly increased (*P* < 0.01), and the expression levels of p-STAT4 and Foxp3 were significantly decreased (*P* < 0.01). Treatment with FAHF-2, or high dose and low dose of GB reversed these changes. Compared with the GBH group, the expression of p-STAT3 and GATA-3 in the GBL group were significantly decreased (*P* < 0.01), and the expression of Foxp3 and p-STAT4 were significantly increased (*P* < 0.01). Compared with the GBL group, the expression p-STAT3 in the FAHF group was further decreased (*P* < 0.01), whereas no statistically significant differences were observed between the two groups for p-STAT4, p-STAT6, Foxp3, or GATA-3.

### Effect of GB intervention on mTOR signaling pathway protein in spleen cells of mice

**Figure 7:**
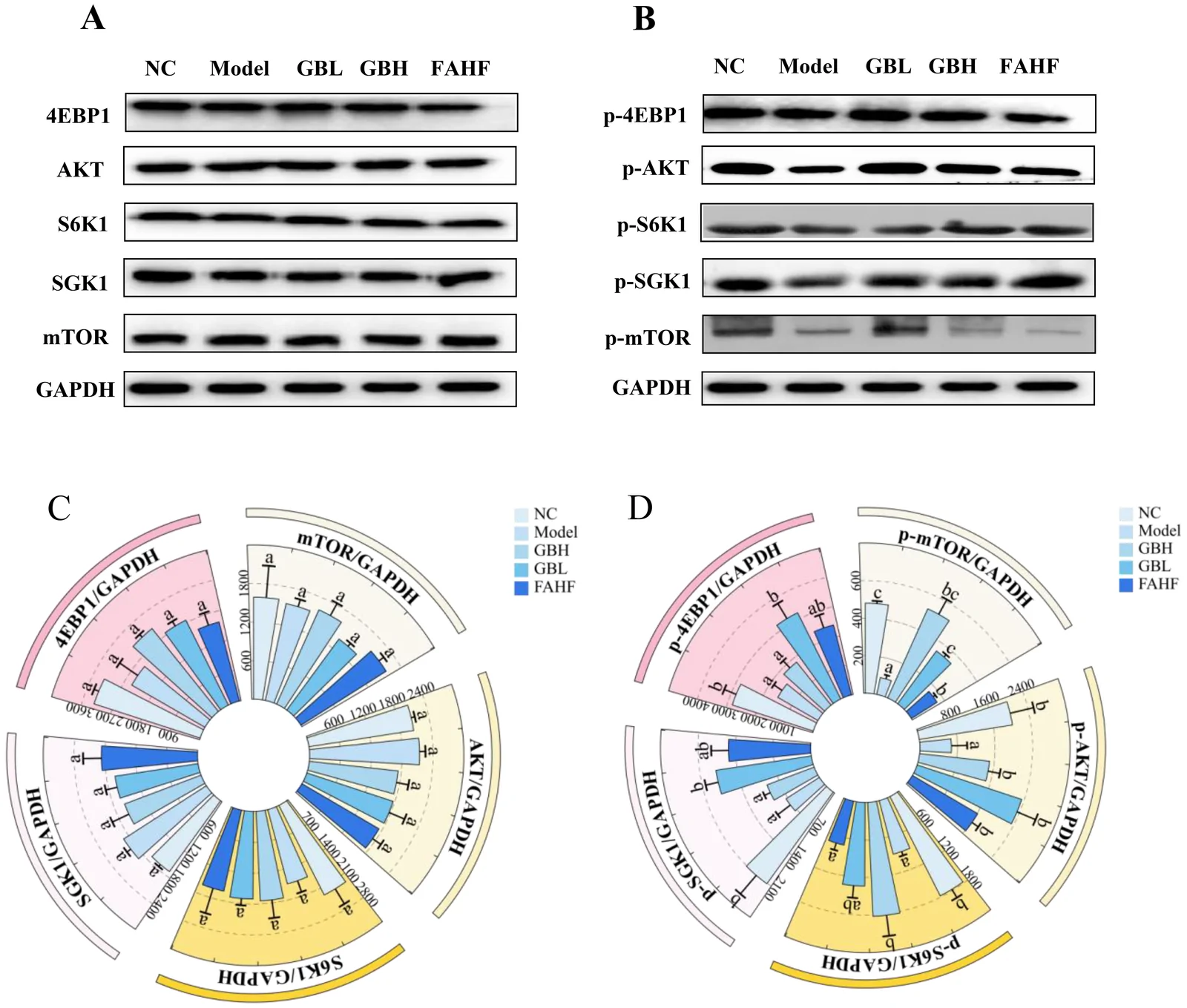
Detection of mTOR-related protein in mouse spleen cells. **(A)** Protein bands of mTOR, AKT, S6K1, SGK1, and 4E-BP1 in each group. **(B)** Protein bands of p-mTOR, p-AKT, p-S6K1, p-SGK1, and p-4E-BP1 in each group. **(C)** Expression of total protein mTOR, AKT, S6K1, SGK1, and 4E-BP1 in mouse splenocytes. **(D)** Expression of protein p-mTOR, p-AKT, p-S6K1, p-SGK1, and p-4E-BP1 in mouse splenocytes (*n* = 5, $\bar{x}$ ± SEM). Different lowercase letters within identically colored fan sectors in **(C, D)** indicate significant differences at *P* < 0.05.

Western Blot was used to detect the expression of mTOR and its downstream proteins AKT, p-AKT, SGK1, p-SGK1, S6K1, p-S6K1, 4EBP1, and p-4EBP1, as shown in **Figure 7**. Compared with the NC group, there was no significant difference in the expression of mTOR and its downstream total protein in the experimental groups (**Figure 7C**). The expression of p-mTOR, p-AKT, p-SGK1, p-S6K1 and p-4EBP1 in the model group was significantly decreased (**Figure 7D**). Compared with the model group, the protein expression of p-mTOR and p-AKT in the three treatment groups were significantly increased. While for the p-SGK1 and p-4EBP1, only the GBL group were increased compared with the model group. Furthermore, compared with the GBH group, the expression of p-SGK1 and p-4EBP1 were increased for the GBL group, but not the FAHF group.

## Discussion

It is well-established that more than 90% of food allergic reactions are caused by eight major food categories: eggs, peanuts, dairy products, sesame, wheat, nuts, shellfish (including crustaceans and mollusks) and fish (26). Allergens in eggs mainly exist in egg white, in which ovalbumin, ovomucoid, ovotransferrin and lysozyme are commonly recognized (27). This study used egg white in combination with cholera toxin adjuvant to establish a food allergy model in BALB/c mice.

When allergens induce susceptible individuals to produce enough specific IgE to bind with receptors on the surface of mast cells and basophils, the body comes to a sensitized state. Upon subsequent re-exposure to the same allergens, crosslinking of membrane-bound IgE triggers cellular degranulation and initiates allergic reactions (28). Therefore the change of serum specific IgE level and allergic responses are key criteria for evaluating food allergy. In this experiment, after the construction of the mice food allergy model, the serum IgE levels in food-allergic mice were significantly higher than that of the NC group, which was consistent with the allergic symptom score, indicating that the food allergy mice model was successfully constructed.

In addition to specific IgE, IgG and its subclasses are frequently used as indicators of the severity of food allergy, as certain IgG subclasses, like IgE, can bind to mast cell receptors and influence degranulation and allergic symptoms–for example, Jin et al. (29) reported that the common food preservative tBHQ strongly increased the IgE level in ovalbumin-sensitized mice, and the serum IgG1 level was also increased. Similarly, Samadi et al. (30) reported that the mice model constructed with ovalbumin showed increased IgE, IgG1, and IgG2a levels. Previous research has established that sequential class switching via IgG1 constitutes an important pathway for IgE generation for patients with food allergy, and high IgG1 titers correspond to aggravated Th2 sensitization (31). As reported by Khodoun et al. (32), IgG2a is also capable of driving anaphylaxis. When IgE-mediated anaphylaxis was abrogated in mice, IgG2a alone could still trigger anaphylactic reactions, highlighting its vital involvement in the pathogenesis of food allergy. In our untreated allergic control group, all four antibody isotypes (IgE, IgG, IgG1, and IgG2a) were significantly overproduced, accompanied by severe anaphylactic symptoms, T cell subtype imbalance, and intestinal inflammatory infiltration. After 35 days of GB intervention, declines in IgE, IgG, IgG1, and IgG2a were consistently observed across all treatment groups, which tightly correlated with alleviated allergic manifestations and attenuated intestinal lesions. This uniform reduction of allergen-driven hyperactive humoral antibodies demonstrates the normalization of overactivated Th1/Th2 dual axes rather than generalized immune suppression in SIT (33). The suppressive effect was most prominent in the GBL and FAHF groups, demonstrating that GB treatment at 5 mg/kg/day effectively ameliorated food allergic responses, with an effect comparable to that of the positive standard control.

Food allergy can affect multiple systems such as the digestive tract, skin, and respiratory tract, and as the symptoms relating to the digestive tract are most common, the small intestine was selected for histological evaluation. The intestinal mucosal villi of mice in the model group exhibited marked swelling, shedding, and inflammatory cell infiltration, indicating that food allergy induces intestinal inflammation, which is in accordance with the work of Chen et al. (34). The intestinal inflammatory responses of mice in the GBL, GBH, and FAHF groups were significantly improved, with the most pronounced improvements observed in the GBL and FAHF groups. The improvement of intestinal inflammatory response and villous height in the GB intervention groups suggest that GB effectively alleviates egg-white-induced food allergy in mice. Moreover, for the GBL group, the alleviation efficiency was similar to the FAHF group.

The pathogenesis of food allergy is complex, and it is widely accepted that the imbalances of Th1/Th2 ratio and Treg/Th17 ratio contribute to its development—for example, Wang et al. (35) found that hyperbolic methoxycurcumin can significantly reduce the inflammatory response of intestinal tissue in ovalbumin-allergic mice, reduce the levels of Th2 cytokines IL-4, IL-5, and IL-13, and increase the production of Th1 cytokine IFN-γ in peripheral blood. Kim et al. (36) conducted sublingual immunotherapy in 54 children with peanut allergy and found that, after 12 months, the skin prick wheal size became smaller, and Th2 cytokines IL-4 and IL-13 all significantly decreased. Yin et al. performed a case–control study (20 pairs, each consisting of a child with food allergy and a healthy control) and found that for children with food allergy, the salivary IL-17A and IFN-γ levels were elevated. In the present study, after GB and FAHF-2 intervention, the IL-4, IL-13, and IL-17A levels were all decreased compared with the model group, especially for the GBL and the FAHF group, indicating that GB at a dose of 5 mg/kg/day and FAHF-2 have a better effect on food allergy than GB at a dose of 40 mg/kg/day.

To further validate the spleen cytokine results, flow cytometry was employed to assess the T cell subtypes in mice spleen. The nature of impaired immune response in food allergy lies in the shift from a Treg cellular response to a Th2 dominant cellular response. When the allergens are contacted again after sensitization, a robust Th2 cell immune effector response will occur. Th2 and its derived cytokines drive the typical IgE-dependent allergic pathway, which promotes further Th2 differentiation and inhibits Treg cell function, ultimately leading to the release of mast cell mediators; Treg cell can, in turn, inhibit Th2, affecting B cell differentiation into IgE-producing plasma cells (37). The results in this work showed that after GB intervention the number of Th1 cells and Treg cells were increased, and Th2 and Th17 cells were decreased, exhibiting improved Th1/Th2 and Treg/Th17 ratios, especially for the GBL group, indicating a better therapeutic effect. The flow cytometry indexes also indicated a similar alleviation effect of the GBL and FAHF group.

Signal transducers and activators of transcription (STATs, including STAT1, STAT2, STAT3, STAT4, STAT5a, STAT5b, and STAT6) regulate numerous processes essential for homeostasis and development in mammals and are key turning points in multiple signaling pathways (38). Upon cellular stimulation, STAT proteins become activated via phosphorylation, entering the nucleus to bind to specific DNA sequences, where they affect the transcription of target genes, thereby participating in various biological processes such as T cell proliferation and differentiation—for example, STAT3 promotes the differentiation of Th2 cells and the transformation of Treg cells into Th17 cells; STAT4 is involved in Treg gene transcription; and STAT6 impairs the stability and function of Treg (39–41). Moreover, STAT6, together with GATA3, was found to facilitate the Th2-mediated immunity (42). In this experiment, after treatment with FAHF-2 or high dose and low dose of GB, the expression levels of p-STAT3, p-STAT6, and GATA-3 were decreased, and the expression levels of p-STAT4 and Foxp3 were significantly increased, especially for the low dose of GB, indicating that the therapeutic effect of GB and FAHF-2 may relate to the STAT signaling pathway.

The mammalian/mechanistic target of rapamycin (mTOR) is a multi-nodal signaling pathway that plays a key regulatory role in immune responses (43). The mTOR contains two complexes, mTOR complex 1 (mTORC1) and mTOR complex 2 (mTORC2), which accept different upstream signals and differentially regulate downstream physiological functions: mTORC1, composed of mTOR, Raptor, and mLST8, exerts its effects on protein synthesis promotion by phosphorylating two key effectors, S6K1 and 4EBP1. In contrast, mTORC2 mainly regulates the arrangement of actin cytoskeleton, phosphorylating two important substrates, AKT and SGK1 (44). mTORC1 activation was found to be necessary for IL-12-STAT4 signaling, which is essential for Th1 cell commitment (45). Treg cells also depend on mTORC1 activation to maintain proper differentiation and function, and inhibition of the mTORC2 signaling pathway results in suppressive function of Treg cells (46, 47). In this study, the expression of p-mTOR, p-AKT, p-SGK1, and p-4EBP1 in the GBL group all reversed sensitization-induced changes, suggesting that the mTOR signaling pathway may intervene in food allergy by acting at the source of immune cell differentiation.

An interesting phenomenon observed in this work was that the levels of p-mTOR and p-AKT in the model group were significantly decreased, which contrasts with the expected overactivation of the mTOR pathway in common allergy models. The decreased p-AKT and p-mTOR in our allergic model can be attributed to model duration, immune negative feedback, and detected tissue specificity: Unlike acute short-term allergic models with transient mTOR hyper-activation, our model involves chronic sensitization. Long-term continuous allergen stimulation triggers compensatory immunosuppressive feedback in central splenic immune cells, inhibiting AKT/mTOR phosphorylation to restrict persistent Th2 inflammation. Moreover, we detected changes in the spleen instead of the lung tissue, jejunum, or peripheral blood mononuclear cells commonly focused in other works. Consistent with our results, several studies have reported suppressed AKT/mTOR signaling in splenic immune cells under chronic inflammatory conditions (48).

The results in this work indicated that GB at 5 mg/kg/day was more effective than GB at 40 mg/kg/day. It looks like an unusual phenomenon, but it sometimes happens—for example, Yang et al. (49) found that low doses of dopamine D1 agonists improve working-memory-related behavior, but high doses eliminate the improvement. At the high daily dose of 40 mg/kg/day, sustained over-saturation of target receptors occurs. Continuous excessive ligand binding triggers cellular adaptive feedback: receptors are internalized and degraded, or their downstream signaling pathways are desensitized (49–51). Even with abundant drug molecules, cells lose responsiveness to stimulation. These might have caused the effect of 40 mg/kg/day to be not as good as that of 5 mg/kg/day. We did not explain this phenomenon as being due to toxicity because the acute intravenous injection toxicity study indicated that the LD_50_ value of GB was 428.6 mg/kg/day, and at 200 mg/kg/day GB it still showed a protective effect on the kidney of mice (52, 53). The therapeutic doses adopted in this work are far below this toxic threshold, confirming that the tested GB dosages possess a broad safety margin.

One limitation of the present study is that it only focuses on the phenotypic change of mTOR phosphorylation induced by GB in splenic immune cells under allergic conditions and cannot fully distinguish direct vs. indirect regulatory effects. Follow-up experiments will be carried out to further identify the exact upstream target of GB.

## Conclusion

The current research has identified a potential active ingredient in *Ginkgo biloba* for the treatment of food allergy. It has therapeutic efficacy comparable to that of the reported best TCM formula FAHF-2. Furthermore, our findings suggest that the mechanism of GB intervention in food allergy may involve the STAT signaling and the mTOR signaling pathway. Further research will focus on the specific role of pathway proteins in the allergy treatment process.

## Data Availability

The raw data supporting the conclusions of this article will be made available by the authors, without undue reservation.
